# Deletion of Apoptosis Signal-Regulating Kinase 1 (ASK1) Protects Pancreatic Beta-Cells from Stress-Induced Death but Not from Glucose Homeostasis Alterations under Pro-Inflammatory Conditions

**DOI:** 10.1371/journal.pone.0112714

**Published:** 2014-11-10

**Authors:** Emilie Pepin, Arisa Higa, Carole Schuster-Klein, Catherine Bernard, Thierry Sulpice, Beatrice Guardiola, Eric Chevet, Thierry Alquier

**Affiliations:** 1 Betagenex Inc, Laval, QC, Canada, H7V5B7; 2 Inserm U1053, Team Endoplasmic Reticulum Stress and Cancer, Université de Bordeaux, Bordeaux, France, 33076; 3 Physiogenex SAS, Labège, France, 31670; 4 Groupe de Recherche Servier, Suresnes, France, 92284 Cedex; 5 Montreal Diabetes Research Center, Centre de Recherche du Centre Hospitalier de l’Université de Montréal (CRCHUM), and Departments of Medicine, Biochemistry and Cell Biology and Pathology, Université de Montréal, Montréal, QC, Canada, H3T 1J4; The University of Tokyo, Japan

## Abstract

**Background:**

Type 2 diabetes is characterized by pancreatic beta-cell dysfunction and is associated with low-grade inflammation. Recent observations suggest that apoptosis signal-regulating kinase 1 (ASK1) is involved in beta-cell death in response to different stressors. In this study, we tested whether ASK1 deficiency protects beta-cells from glucolipotoxic conditions and cytokines treatment or from glucose homeostasis alteration induced by endotoxemia.

**Methodology/Principal Findings:**

Insulin secretion was neither affected upon shRNA-mediated downregulation of ASK1 in MIN6 cells nor in islets from ASK1-deficient mice. ASK1 silencing in MIN6 cells and deletion in islets did not prevent the deleterious effect of glucolipotoxic conditions or cytokines on insulin secretion. However, it protected MIN6 cells from death induced by ER stress or palmitate and islets from short term caspase activation in response to cytokines. Moreover, endotoxemia induced by LPS infusion increased insulin secretion during hyperglycemic clamps but the response was similar in wild-type and ASK1-deficient mice. Finally, insulin sensitivity in the presence of LPS was not affected by ASK1-deficiency.

**Conclusions/Significance:**

Our study demonstrates that ASK1 is not involved in beta-cell function and dysfunction but controls stress-induced beta-cell death.

## Introduction

Type 2 diabetes (T2D) results from a combination of diminished insulin sensitivity in peripheral tissues and of defective insulin secretion by the pancreatic beta-cell. T2D is considered as a state of chronic and low-grade inflammation and increased levels of interleukin-1β (IL-1β), IL-6 and C-reactive protein are predictive of T2D [1]. It is generally accepted that this inflammatory state is induced by nutrient excess and overweight but recent evidence also suggests that alterations of the gut microbiota in T2D patients leads to increased circulating levels of lipopolysaccharides (LPS) and low grade endotoxemia (reviewed in [2]). In turn, chronic endotoxemia and the associated inflammation alter glucose metabolism and insulin sensitivity in peripheral tissues. This concept is supported by several pieces of evidence. First, endotoxemia induces inflammation and insulin resistance in rodents and humans [3]–[8]. Second, manipulation of the gut microbiota reduces circulating LPS levels and protects from diet-induced glucose intolerance, insulin resistance and inflammation [9]–[12]. Finally, loss of function of the LPS receptor Toll-like receptor 4 (TLR4) [13]–[16] or of its co-receptor CD14 [3] prevents insulin resistance during diet-induced obesity or induced by fatty-acids.

In addition to the deleterious effect of LPS on insulin sensitivity, several studies have established that both LPS and inflammatory cytokines target pancreatic beta-cells, thereby leading to altered glucose-stimulated insulin secretion (GSIS) and to beta-cell death. LPS impairs glucose-stimulated insulin secretion (GSIS) and decreases insulin gene expression in beta-cell lines and isolated rodent islets in a TLR4-dependent manner [17]–[19]. Similarly, pro-inflammatory cytokines, including IL-1β and Tumor necrosis factor-alpha (TNFα), produced by activated macrophages [20], adipocytes and also by pancreatic beta-cells [21] induce beta-cell dysfunction and apoptosis. Importantly, high glucose and saturated fatty acids induces the production of proinflammatory cytokines and chemokines by beta-cells and islet resident macrophages thus creating a vicious cycle by which metabolic and inflammatory disorders impair beta-cell function which in turn further aggravates metabolic perturbations (reviewed by [22]). Hence, nutrient excess, endotoxemia and the associated pro-inflammatory cytokines have deleterious effect on both insulin sensitivity and beta-cell function, which may contribute to the etiology of T2D.

The deleterious effects of saturated fatty acids, LPS and cytokines such as IL-1β on beta-cell function involve the activation of stress pathways consisting of the endoplasmic reticulum (ER) stress [23], [24], the TLR4-signaling pathway [19], [25], the transcription factor nuclear factor kappa-light-chain-enhancer of activated B cells (NF-κB) [19], [26] and the mitogen-activated protein kinase (MAPK) signaling pathway [24], [27].

The MAPK pathway includes c-jun NH2-terminal kinase (JNK) and p38 MAPK, which are both activated by IL-1β in islets and insulinoma cells [24]. Importantly, either pharmacological inhibition or loss of function of JNK and p38 decreases the deleterious effect of IL-1β on insulin secretion, insulin gene transcription and beta-cell apoptosis [24], [28], [29]. However, little is known regarding the upstream kinases responsible for JNK and p38 activation in response to pro-inflammatory cytokines in beta cells.

MAPK activation is triggered by MAPK kinases, which are themselves activated by MAPK kinase kinases (MAPKKK). Apoptosis signal-regulating kinase 1 (ASK1), which is encoded by *Map3k5*, is a member of the MAPKKK family known to activate JNK and p38 in response to various stresses including oxidative stress, ER stress, cytokines and LPS in immune cells and neurons (reviewed by [30]). Importantly, activation of the ASK1-p38 pathway is required for LPS-induced inflammatory responses in immune cells [31]. Moreover, ASK1-deficient mice are resistant to LPS-induced septic shock thereby demonstrating that ASK1 plays a pivotal role in innate immunity and inflammation induced by LPS [31].

Interestingly, recent data have shown that ASK1 is activated by ER stress in MIN6 cells [32]. In addition, ASK1 deficiency *in vivo* decreases beta-cell apoptosis and delays the onset of diabetes in Akita mice thus suggesting that ASK1 activation in response to stress contributes to beta-cell failure and apoptosis [32]. In line with this idea, it was shown that the protective effect of glucose-dependent insulinotropic polypeptide on beta-cell apoptosis induced by staurosporine is dependent on the inhibition of ASK1 activity in beta-cell line and isolated islets [33]. Altogether, these findings suggest that ASK1 may play an important role in beta-cell failure induced by different stressors. However, the role of ASK1 in beta-cell function and dysfunction induced by nutrient excess, LPS or pro-inflammatory cytokines has not been investigated. In addition, whether ASK1 is part of the mechanisms involved in insulin resistance induced by endotoxemia is unknown. Therefore, the goal of the present study was to determine, using MIN6 cells stably expressing a shRNA directed against ASK1 and ASK1 Knock-Out (KO) mice, whether ASK1 is involved in beta-cell dysfunction under glucolipotoxic and cytokines treatment *in vitro* as well as alterations of insulin secretion and sensitivity in response to LPS *in vivo*.

## Results

### ASK1 deficiency does not affect insulin secretion in islets

**Figure 1:**
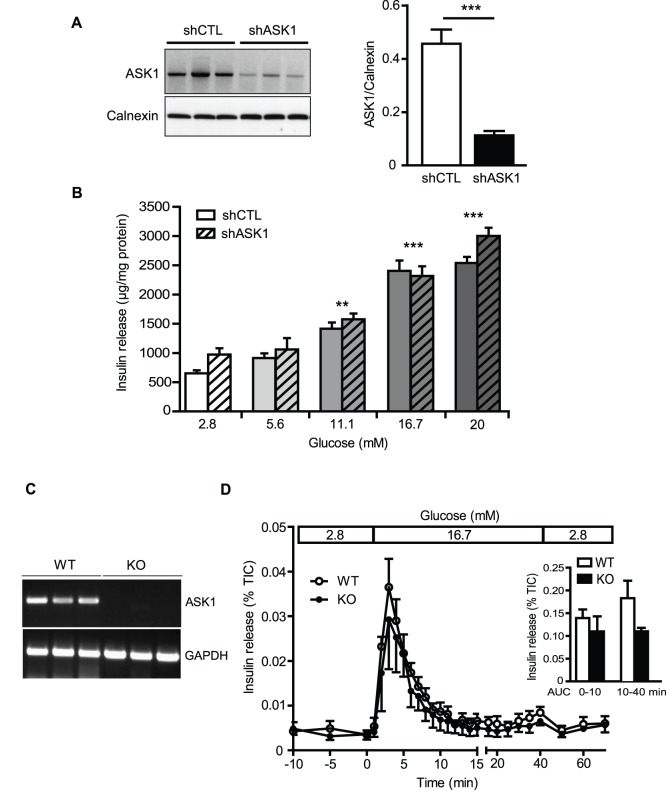
GSIS is not affected by ASK1 deficiency. (A) ASK1 protein levels in MIN6 cells stably expressing a control (shCTL) or shRNA directed against ASK1 (shASK1). (B) Insulin secretion in response to 2.8, 5.6, 11.1, 16.7 and 20 mM glucose in MIN6 shCTL and shASK1cells. **p<0.01 and ***p<0.001 compared to 2.8 mM glucose. (C) ASK1 gene expression assessed by RT-PCR in the liver of WT and ASK-1 KO mice. (D) Insulin secretion in response to 2.8 mM glucose from −10 to 0 min, 16.7 mM from 0 to 40 min and 2.8 mM glucose from 40 to 70 min in WT (open circle) or ASK1-KO (closed circle) islets in perifusion experiments. Insulin release was normalized by total insulin content (TIC). Results are means ± SEM of 3 to 5 independent experiments.

To explore the potential impact of ASK1 deficiency in beta-cell function, GSIS was measured in both MIN6 cells stably expressing a control shRNA (shCTL) or directed against ASK1 (shASK1) (Fig. 1A and B), and in pancreatic islets isolated from wild type (WT) and ASK1-knock out (KO) mice (Fig. 1C). ASK1 shRNA led to a 75% reduction of ASK1 expression compared to MIN6 shCTL cells (Fig. 1A). ASK1 downregulation in MIN6 cells did not affect the dose-response to glucose (Fig. 1B). As expected, the expression of ASK1 was undetectable in the liver of ASK1-KO animals (Fig. 1C). In line with the results obtained in MIN6 cells, we observed that first and second phase of GSIS were similar between WT and ASK1-KO islets in perifusion conditions (Fig. 1D). These results indicate that ASK1 deficiency does not affect insulin secretion in MIN6 cells and isolated islets.

### ASK1 deficiency does not protect from glucolipotoxicity- or cytokines-induced beta-cell dysfunction

**Figure 2:**
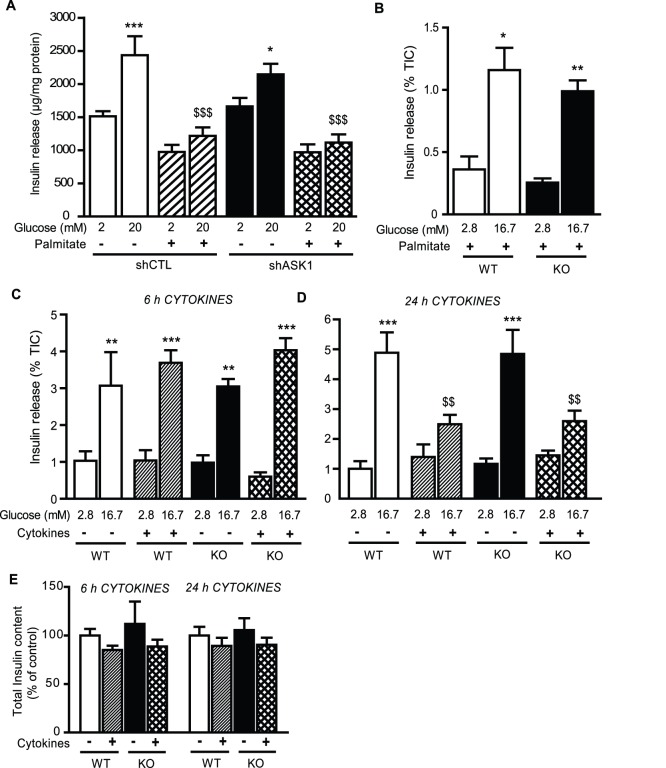
ASK1 deficiency does not protect from palmitate- or cytokines-induced beta-cell dysfunction. (A) Insulin secretion in response to 2 and 20 mM glucose in MIN6 shCTL and shASK1cells pre-incubated during 24 h with 20 mM glucose +/− 0.33 mM palmitate. (B) Insulin secretion in response to 2.8 and 16.7 mM glucose in WT and ASK1-KO islets pre-incubated during 24 h with 16.7 mM glucose +/− 0.5 mM palmitate. Insulin secretion in response to 2.8 and 16.7 mM glucose in WT and ASK1-KO islets treated with cytokines during 6 h (C) and 24 h (D). (E) Total insulin content in glucose in WT and ASK1-KO islets treated with cytokines during 6 h and 24 h. Results are mean ± SEM of 3 to 6 independent experiments. *p<0.05, **p<0.01 and ***p<0.001 compared to low glucose condition. $$*p*<0.01 and $$$*p*<0.001 compared to high glucose in control conditions.

The combination of high glucose and palmitate, i.e. glucolipotoxic conditions, is well known to alter GSIS in beta-cells and islets (reviewed by [34]). Thus, we tested whether ASK1 downregulation protects beta-cells from alteration of GSIS induced by glucolipotoxic conditions. As expected, treatment with high glucose (20 mM) and palmitate (0.33 mM) for 24 h significantly impaired GSIS in MIN6 cells (Fig. 2A). However, this response was not altered upon ASK1 silencing (Fig. 2A). In agreement with these results, GSIS was similar in WT and ASK1-KO islets incubated in similar glucolipotoxic conditions (Fig. 2B) thereby showing that ASK1 deficiency does not protect from beta-cell dysfunction induced by glucolipotoxic conditions.

Pro-inflammatory cytokines have deleterious effect on beta-cell function and survival [22] and are known to activate ASK1 in immune cells [31]. Thus, we tested whether ASK1 deficiency protects beta-cell from dysfunction induced by cytokines treatment. To this end, GSIS was measured in static incubations of WT and ASK1-KO islets treated with IL-1β (10 units/ml) and IFN-γ (100 units/ml) for 6 and 24 h. First, both basal and GSIS were similar in WT and ASK1-KO islets in control conditions (Fig. 2C and D), in agreement with the perifusion results shown in Fig. 1D. GSIS was not affected by a 6 h cytokine treatment (Fig. 2C). In contrast, 24 h cytokine treatment significantly decreased the ability of glucose to stimulate insulin secretion in WT islets (Fig. 2D). However, the inhibitory effect of cytokines on GSIS was not affected by ASK1 deficiency (Fig. 2D). There was no significant decrease in insulin content in islets isolated from either genotype in response to cytokines after 6 or 24 h (Fig. 2E).

### ASK1 deletion protects beta-cell from stress-induced cell death

**Figure 3:**
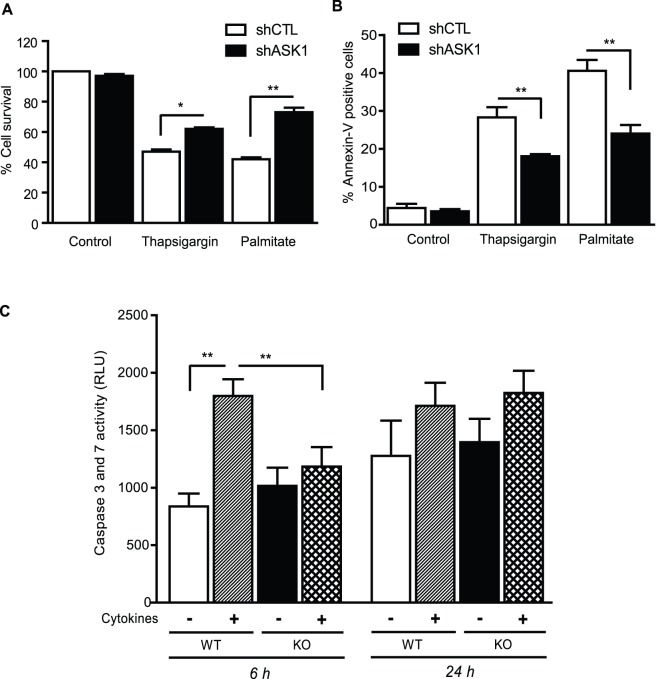
ASK1 deficiency protects from beta-cell death and caspase activation. MIN6 shCTL and shASK1 cells survival (A) and apoptosis (B) in response to thapsigargin (0.5 µM) or palmitate (0.5 mM). (C) Caspase 3 and 7 activities in WT and ASK1-KO islets treated with cytokines during 6 h and 24 h. RLU, relative luminescence unit. Results are mean ± SEM of 4 to 6 independent experiments. *p<0.05 and **p<0.01.

Based on the role of ASK1 in apoptosis [35], we tested whether ASK1 protects from beta-cell death induced by lipotoxicity, ER and inflammatory stress. ER stress was induced by thapsigargin (0.5 µM). Lipotoxicity was induced by palmitate (0.5 mM), a saturated fatty acid known to generate oxidative stress, ceramide and to induce ER stress in beta-cells (reviewed in [34]). In MIN6 cells, ASK1 silencing partially protected beta-cells from apoptosis death induced by thapsigargin and palmitate (Fig. 3A and B). In isolated islets, we observed that pro-apoptotic caspases 3 and 7 activation in response to cytokines was totally prevented by ASK1 deficiency (Fig. 3C) after 6 h. However, caspases activation induced by cytokines was not sustained after 24 h of treatment (Fig. 3C). These results suggest that ASK1 is implicated in the cytotoxic effects of ER stress, palmitate and pro-inflammatory cytokines.

### Insulin secretion and sensitivity under basal conditions or in response to LPS are not affected by ASK1 deficiency

**Figure 4:**
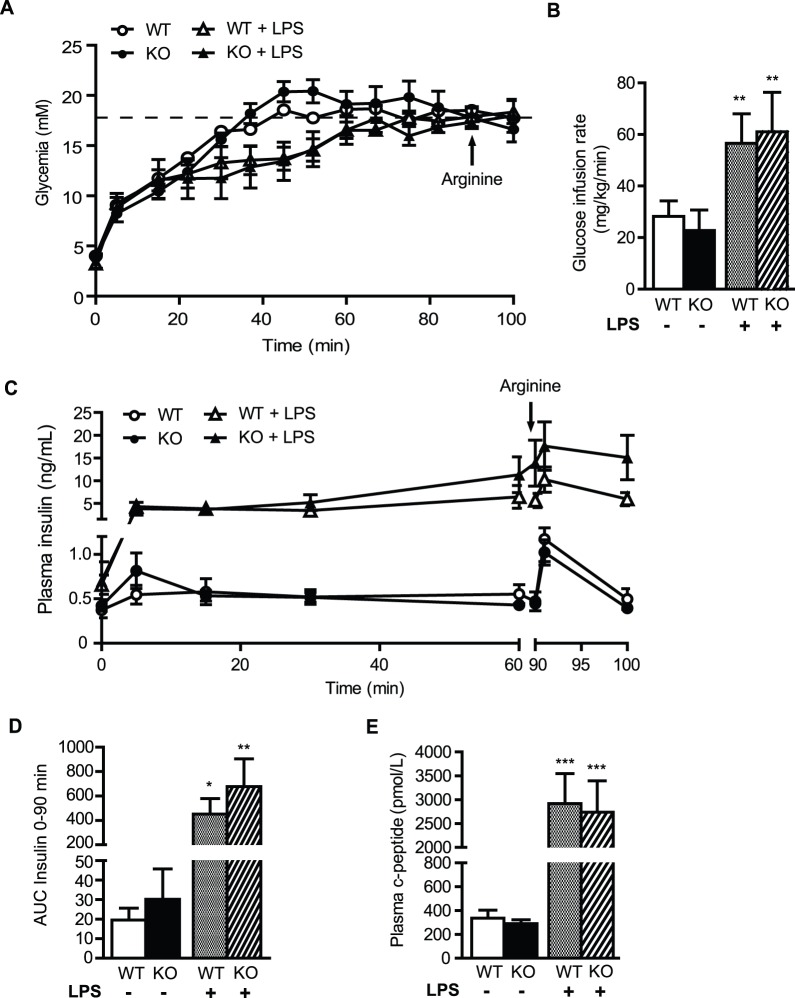
Hyperglycemic clamps in LPS-infused WT and ASK1-KO mice. Glucose (A), glucose infusion rate (GIR 60–90 min) (B) and plasma insulin levels (C) during the course of the hyperglycemic clamp in PBS- and LPS-infused WT and ASK1-KO mice. (D) Area under the curve (AUC) for insulin over the first 90 min of the clamp. (E) C-peptide levels at 75 min. Values are expressed as means ± SEM of 7 to 8 mice per group. *p<0.05, **p<0.01 and ***p<0.001 compared to control condition without LPS for each genotype.

Chronic endotoxemia and inflammation lead to insulin resistance. Given the role of ASK1 in LPS-mediated innate immunity and inflammation, we tested the hypothesis that ASK1 deficiency protects from LPS-induced alteration of insulin sensitivity and beta-cell function *in vivo*. To this end, WT and ASK1-KO mice were infused intravenously with PBS or LPS during 24 h. Since LPS has an anorectic action, all animals (PBS- and LPS-infused) were fasted during the infusion to avoid any confounding effect induced by differences in feeding. At the end of the infusion, insulin secretion in response to glucose and arginine was assessed using hyperglycemic clamps (Fig. 4A and B). In control animals treated with PBS, insulin secretion during the clamp was similar between WT and ASK1-KO mice (Fig. 4C and D). Accordingly, C-peptide levels 75 min into the clamp were not different (Fig. 4E). Arginine-potentiation of GSIS was also similar in WT and ASK1-KO animals (Fig. 4C). In LPS-infused mice, the glucose infusion rate required to reach the target blood glucose during the steady state of the clamp (60–90 min) was significantly higher than in PBS-infused mice (Fig. 4B). GSIS was dramatically increased by ∼10 fold in LPS- vs. PBS-infused animals (Fig. 4C and D). In line with these results, C-peptide levels were significantly higher in WT and ASK1-KO mice treated with LPS (Fig. 4E). However, no difference was observed when comparing GSIS, C-peptide levels and potentiation of GSIS by arginine between WT and ASK1-KO mice.

**Figure 5:**
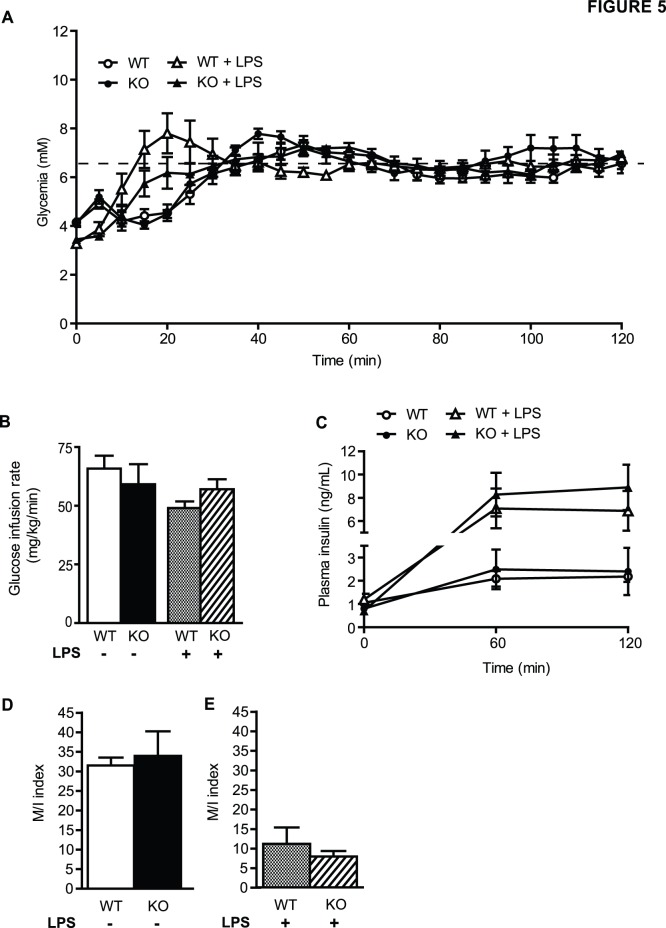
Hyperinsulinemic-euglycemic clamps in LPS-infused WT and ASK1-KO mice. Glucose (A), glucose infusion rate (GIR 60–120 min) (B) and plasma insulin levels (C) during the course of the hyperinsulinemic-euglycemic clamp in ASK1-KO and WT littermates infused with PBS or LPS during 24 h. (D–E) Insulin sensitivity index (M/I). Values are expressed as means ± SEM of 6 to 7 mice per group.

Whole-body insulin sensitivity was measured in hyperinsulinemic-euglycemic clamps in a second group of LPS-infused mice (Fig. 5A). The glucose infusion rate (Fig. 5B) required for maintaining glycemia at 6.5 mM (Fig. 5A) during the clamp was similar in PBS- and LPS-infused animals and was not different in WT vs. ASK1-KO mice. LPS administration dramatically increased circulating levels of insulin by ∼8 fold during the clamp compared to control conditions (Fig. 5C) but this response was not affected by ASK1 deficiency. The dramatic rise in insulin levels in LPS-treated mice, even under clamp conditions, precluded a comparison between LPS-treated and control animals. However, comparing PBS-infused WT vs. ASK1 KO mice on the one hand (Fig. 5D), and LPS-infused WT vs. ASK1 KO mice on the other hand (Fig. 5E) showed no effect of ASK1 deletion on the M/I index of insulin sensitivity.

## Discussion

The objectives of this study were to examine whether deletion of ASK1 protects from the deleterious effect of glucolipotoxic and cytokines-induced stress pathways on GSIS and survival in beta-cells and on alteration of insulin secretion and sensitivity induced by LPS *in vivo*. First, we found that GSIS in MIN6 cells, isolated islets and *in vivo* is not affected by ASK1 deficiency suggesting that ASK1 is not required for the normal response to glucose. In addition, insulin sensitivity during hyperinsulinemic-euglycemic clamps was similar in WT and ASK1-KO mice. Together, these results demonstrate that ASK1 deficiency does not affect insulin secretion and sensitivity *in vivo*. Second, we observed that ASK1 deficiency in MIN6 cells and islets did not prevent beta-cell from dysfunction induced by glucolipotoxic conditions or by cytokines but protected beta-cell from death and short term caspase activation in response to ER, lipotoxic and pro-inflammatory stressors, suggesting a specific involvement of ASK1 in beta-cell death but not beta-cell dysfunction. Third, alteration of insulin secretion and sensitivity induced by LPS systemic infusion was not affected by ASK1 deletion, suggesting that ASK1 is not involved in the glucoregulatory responses to endotoxemia.

Although GSIS was not affected *in vitro* and *in vivo* by ASK1-deficiency, we can not rule out that islet morphology and/or the proportion of alpha- and beta-cells in islets is different in ASK1-KO mice. However, insulin and glucose plasma levels were not different in basal conditions and C-peptide levels were similar in hyperglycemic conditions in WT and ASK-1 KO mice. This suggests that potential changes in alpha- and/or beta-cells mass did not affect glucoregulatory responses. Nevertheless, additional studies will be required to assess the potential effect of ASK1 deficiency on islet cell differentiation and plasticity.

LPS is well known to induce the release of pro-inflammatory cytokines and to profoundly affect glucose homeostasis [3], [5], [7]. Based on *in vitro* studies, it has been suggested that the underlying factors leading to glucose homeostasis impairment include insulin resistance and alteration of GSIS. Indeed, treatment of islets [19], [20] or insulinoma cells with LPS [17], [18] or with cytokines [22] dramatically alters insulin secretion. In agreement with these studies, we found that GSIS was impaired in response to IL-1β and IFN-γ treatment in isolated islets.

During hyperinsulinemic-euglycemic clamps, we observed that LPS infusion lead to ∼8 fold increase in insulin levels not allowing to accurately compare the M/I Index between PBS and LPS-infused animals. Importantly, these results are in agreement with a recent study showing a similar increase in insulin levels during a hyperinsulinemic clamp in response to LPS [7]. Despite this limitation, the fact that the glucose infusion rate required to maintain euglycemia was similar between PBS- and LPS-infused mice in the face of higher insulin levels in LPS treated animals strongly support the notion that LPS decreased insulin sensitivity in our model.

During hyperglycemic clamp, we also found that insulin levels were significantly higher in LPS infused animals. Both insulin and C-peptide levels were increased by ∼8 fold in LPS-infused mice compared to control animals thereby demonstrating that LPS stimulates insulin secretion. These findings are consistent with and confirm a recent study by Nguyen *et al.* showing that acute or chronic LPS administration enhances glucose tolerance by increasing insulin secretion during glucose tolerance tests [36].

To our knowledge, this is the first report showing that LPS increases insulin secretion during hyperglycemic clamp. Taken together, our clamp results suggest that the increased insulin secretion in response to LPS could be a compensatory mechanism to decreased insulin sensitivity. Although it was not measured in the current study, the underlying mechanisms responsible for enhanced insulin secretion by LPS may involve activation of the Glucagon Like Peptide-1 signaling pathway in beta-cells as recently shown by Nguyen *et al.* [36].

Cytokines levels were not measured in response to LPS in WT and ASK1-KO animals, thus it is difficult to determine whether LPS triggered inflammatory responses and whether these responses were affected by ASK1 deficiency in our model. However, previous studies showing that a similar dose of LPS increased cytokines [7], [37] and that LPS-induced inflammation is dependent on ASK1 [31] makes it temping to speculate that LPS lead to pro-inflammatory cytokines secretion and that this response was decreased in ASK1-KO mice. Despite this limitation, ASK1 deficiency did not alter the effect of LPS on insulin secretion and sensitivity demonstrating that ASK1 is not involved in LPS-mediated alteration of glucose homeostasis.

Taken together, our findings do not support the hypothesis that ASK1 regulates beta-cell function or protect beta-cells from dysfunction induced by inflammatory signals such as cytokines or LPS. Previous studies have established that either ASK1 deletion or expression of a dominant-negative ASK1 isoform protects from ER-stress and staurosporine-induced caspase 3 activation and beta-cell apoptosis in insulinoma cells and islets thereby supporting a pivotal role of ASK1 in beta-cell apoptosis in response to stressors [32], [33]. Importantly, ASK1 deficiency delayed the onset of hyperglycemia in Akita mice and decreased the number of apoptotic beta-cells [32]. However, beta-cell function and GSIS were not measured in those studies. Consistent with a role for ASK1 in beta-cell survival, we found that ASK1 deficiency decreased MIN6 cell death in response to the ER stress inducer thapsigargin and to palmitate as well as caspase 3 and 7 short term activation by cytokines in islets. However, the protective effect of ASK1 deletion or silencing did not translate into improvement in GSIS. This combined with the aforementioned studies suggest that the ASK1 pathway regulates beta-cell survival but not function. We must point out that the activation of caspases by cytokines in islets was transient and not sustained after 24 h (Fig. 3C) suggesting that the cytokine treatment at this concentration may not lead to beta-cell death. This is supported by the fact that total insulin content in islets was not affected after 6 and 24 h treatment with cytokines (Fig. 2E). In addition, it is possible that the effect of glucolipotoxic conditions and cytokines on beta-cell function is dependent on the treatment duration. This is exemplified by the fact that cytokines induced a transient activation of caspases in isolated islets (Fig. 3C). Therefore, we cannot rule out that MIN6 shASK1 cells or ASK1-KO islets might be protected against alteration of GSIS after longer period of treatment with cytokines or glucose and palmitate. In the same line, it is possible that long term treatment with LPS *in vivo* might lead to beta-cell dysfunction and highlight phenotypic differences between WT and ASK1-KO mice. Finally, we cannot rule out that ASK1 deficiency is compensated by ASK2 or ASK3 in islets [38]. Similarly, such compensation could explain in part the absence of differential responses to endotoxemia between WT and ASK1-deficient mice.

In summary, we found that ASK1 does not regulate beta-cell function and the deleterious effect of cytokines and glucolipotoxic conditions on insulin secretion. In addition, ASK1 deficiency has no impact on the changes in glucose homeostasis in response to LPS. However, ASK1 deficiency reduces beta-cell death induced by stress pathways, suggesting that ASK1 regulates beta-cell survival but not function. Whether or not ASK1 is involved in the etiology of beta-cell failure and hyperglycemia in T2D rodent models will require further investigations.

## Methods

### Reagents

DMEM, RPMI-1640, fetal bovine serum (FBS) and antibiotics were obtained from Invitrogen (Burlington, ON, Canada). PBS was obtained from Multicell. Fatty acid-free bovine serum albumin (BSA) was from Equitech-Bio (Kerrville, TX, USA). The cytokine cocktail (IL-1β and IFN-γ) was from R&D Systems (Minneapolis, MN, USA). Antibodies to ASK1 and calnexin were from Abcam (Cambridge, MA, USA). LPS (from Escherichia coli O55:B5) and all other reagents (analytical grade) were from Sigma unless otherwise noted.

### Animals

ASK1 knock-out (KO) mice were obtained from Dr Hidenori Ichijo (Tokyo, Japan) [35]. Animals were housed on a 12-h light/dark cycle at 21°C with free access to water and standard chow diet. ASK1 heterozygous knockout (Het) mice were bred to generate experimental male ASK1 wild-type (WT) and homozygous KO. Animals were euthanized by exsanguination under ketamine/xylazine anesthesia. All procedures using animals were approved by the institutional animal care and use committee (CIPA, protocol #An11015VPs).

### Plasmid construction, MIN6 infection and selection

Two shRNA lentiviral plasmids were used to silence ASK1 in MIN6 cells [39]. We designed shRNA sequences against mouse ASK1 (GTGAAGTTTCATTACGCAT) that were cloned into the pLVTHM vector downstream the H1 promoter. To this end, the double-strand fragment was generated by the annealing of the two complementary oligonucleotides (see above) flanked by the MluI and ClaI restriction sites. The pLVTHM was digested with CLaI and MluI and the corresponding fragments ligated using DNA ligase. Plasmids were amplified and sequence verified (using a H1promoter primer) prior to being packaged in the appropriate system. MIN6 cells were infected with the lentivirus pool (using a luciferase (GL2) targeting shRNA plasmid as control) and the population expressing GFP sorted by FACS during 5 generations leading to the selection of a stably silenced homogeneous population. The GFP expressing populations were evaluated for ASK1 expression using Western blot with an anti-ASK1 antibody (Abcam, ab62575; dil. 1/1000). ASK1 shRNA led to a significant 75% extinction of ASK1 expression (Fig. 1A).

### ASK1 mRNA expression by RT-PCR

ASK1 mRNA levels were quantified in the liver of WT and ASK1-KO mice using RT-PCR. Total RNA were extracted using Trizol (Invitrogen Life Technologies, Carlsbad, CA) and reverse-transcribed as previously described [40]. The first-strand complementary DNA was synthesized using SuperScript 2 (Invitrogen Life Technologies) and cDNA was amplified by PCR using ASK1 forward primer CCTGAAGCTTAAGTCCCAACC and reverse GCATCCCTCCCCTTAGTCTC. Glyceraldehyde-3-phosphate dehydrogenase (GAPDH) was amplified as an internal control using forward primer GGTGAAGGTCGGAGTCAACGGA and reverse GAGGGATCTCGCTCCTGGAAGA.

### Cell culture

MIN6 shCTL and shASK1 cells were grown in DMEM containing 25 mM glucose supplemented with 15% FBS, 100 µg/ml streptomycin and 100 units/ml penicillin sulfate at 37°C in a humidified 5% CO_2_ atmosphere. Culture media was changed every 2 to 3 days of culture. For insulin release experiments, cells were plated in 24-well plates at a density of 0.2×10^6^ cells per well for 3 days.

### Insulin Secretion in MIN6 cells

Insulin secretion was measured in static conditions at 37°C. After a 30 min starvation in DMEM without glucose, insulin secretion was measured during 2 h in HEPES-balanced Krebs-Ringer bicarbonate buffer (125 mM NaCl; 5.9 mM KCl; 1.2 mM MgCl_2_; 1.3 mM CaCl_2_; 25 mM HEPES, pH 7.4) containing 0.1% BSA and different glucose concentrations. At the end of incubation, insulin concentration was measured in the medium using a mouse insulin Elisa kit (Eurobio, Courtaboeuf, France) and normalized by cell protein content.

### MIN6 cells in glucolipotoxic conditions

Palmitate was dissolved in 50% ethanol to prepare 100 mM stock solutions. Three days after seeding, MIN6 shCTL and shASK1 cells were cultured for 24 h in DMEM 25 mM glucose, 0.5% BSA with or without 0.33 mM palmitate. Glucose-induced insulin secretion was then measured in static conditions during 30 min as described in the previous section.

### MIN6 cells toxicity assay

MIN6 shCTL and shASK1 cells were seeded into 96-well plates at a density of 5000 cells/well. Plates were incubated at 37°C with 10% CO2 for 24 h prior to addition of experimental drugs. After 24 h, a plate of each cell line was fixed in situ with trichloroacetic acid and sulforhodamine B staining to provide a measurement of the cell population for each cell line at the time of drug addition. Cells were incubated with or without drugs (0.5 µM Thapsigargin for 15 h and 0.5 mM palmitate complexed to BSA for 48 h). The assay was terminated by the addition of cold trichloroacetic acid; sulforhodamine B staining was performed, and absorbance was measured at 510 nm. Cell growth was evaluated at each drug concentration by using the following formula (Ti−Tz)/(C−Tz)×100 where Ti represents the absorbance of the treated cells and C the absorbance of the untreated (control) cells.

### MIN6 cells apoptosis assay

Flow cytometry-based analysis of cell apoptosis was performed following staining of the cells with Annexin V-FITC and propidium iodide (PI) using the Annexin-V FITC kit (Annexin-V FITC Apoptosis Kit, Beckman Coulter) according to the manufacturer’s protocol and as previously described [40]. Thapsigargin (0.5 µM) - and palmitate (0.5 mM)-induced apoptosis was assessed using the following formula: percent specific apoptosis = (test−control)×100/(100−control). The extent of apoptosis was quantified as the percentage of Annexin V–positive cells as previously described [40].

### Islet isolation and culture

Islets were isolated from 10- to 14-week old animals by collagenase digestion and handpicking under a stereomicroscope as previously described [41] and cultured for 1 h (Fig. 1) in RPMI-1640 supplemented with 10% (wt/vol.) FBS, 100 U/ml penicillin–streptomycin and 11 mM glucose or in the same media supplemented with a cocktail of cytokines consisting of 10 units/ml of Interleukin-1β and 100 units/ml of Interferon-γ (Fig. 2 and 3) during 6 or 24 h.

### Islets perifusions and static incubations

Islet perifusions were performed as described [42]. Briefly, batches of 150 islets each were placed in Swinnex chambers (Millipore, Nepean, ON, Canada) and perifused for a total of 80 min with KRB buffer containing 0.1% (wt/vol.) BSA and 2.8 mM glucose. After a 10-min equilibration period with KRB solution with 2.8 mM glucose, islets were perifused for 40 min with 16.7 mM glucose. At 40 min the glucose concentration of the KRB was decreased to 2.8 mM. Intracellular insulin was extracted with acidified ethanol at the end of the perifusion to measure insulin content. For static incubations, batches of ten islets each were starved twice in KRB solution containing 0.1% (wt/vol.) BSA and 2.8 mM glucose for 20 min at 37°C then incubated for 1 h in the presence of 2.8 or 16.7 mM glucose. Each condition was run in triplicate. Intracellular insulin content was measured after acid–ethanol extraction. Insulin was measured by radioimmunoassay using rat insulin RIA kit (Millipore, Billerica, MA, USA).

### Caspase activity

Batches of 150 islets were cultured in presence of 10 units/ml of Interleukin-1β and 100 units/ml of Interferon-γ during 6 or 24 h. Caspase-3 and-7-like activity was measured by a luminescent assay kit (Caspase-Glo 3/7 Assay, Promega), in which a synthetic peptide Z-DEVD-aminoluciferin was used as a substrate, following the manufacturer’s instructions. The luminescence of the aminoluciferin released was measured with a plate-reading luminometer.

### LPS infusion

A LPS solution (0.04 mg/ml diluted in sterile PBS) was infused through the jugular vein at a rate of 0.042 mg/kg/h during 24 h for a total of 1 mg/kg (1 µl/h/g). All animals were fasted during the 24 h infusion to avoid the confounding effect of LPS on food intake. Control animals were infused with a similar volume of sterile PBS. LPS and PBS infusion was stopped right before the beginning of the hyperglycemic and euglycemic-hyperinsulinemic clamp.

### Assessment of insulin secretion and sensitivity by hyperglycemic and euglycemic-hyperinsulinemic clamp

One-step hyperglycemic clamps were performed on conscious animals as described [41]. A 20% dextrose solution was infused through the jugular vein to clamp plasma glucose at 18 mM for 100 min and was adjusted based on glucose measurements (Roche Accu-Check; Roche, Indianapolis, IN). At 90 min, an arginine bolus injection was performed (1 mmol/kg; Sandoz Canada) to assess the maximal insulin response. Plasma samples were collected from the tail at several time points during the clamp for insulin measurements using a mouse insulin enzyme-linked immunosorbent assay (ELISA) kit (Alpco Diagnostics, Salem, NH). Plasma samples for C-peptide measurements were collected at 75 min and analyzed using a mouse C-peptide ELISA kit (Alpco Diagnostics). Two-hour hyperinsulinemic-euglycemic clamps were performed in ASK1 wild-type and knockout mice as previously described [41]. Briefly, following a 1-min bolus insulin infusion (85 mU/kg; Humulin R), insulin was infused at 5 mU/kg/min. Twenty percent dextrose was infused starting 5 min after the insulin infusion to clamp glycemia at ∼6.5 mM. The insulin sensitivity index (*M*/*I*) was calculated as the glucose infusion rate (*M*) divided by the average insulinemia during the last 60 min of the clamp (*I*).

#### Expression of data and statistics

Data are expressed as means ± SEM.

Intergroup comparisons were performed by ANOVA, with post hoc adjustments for two-by-two comparisons or Student’s *t* test, as appropriate. *P*<0.05 was considered significant.
